# PPAR*α* Regulates the Proliferation of Human Glioma Cells through miR-214 and E2F2

**DOI:** 10.1155/2018/3842753

**Published:** 2018-05-14

**Authors:** Yong Gao, Dongfeng Han, Laisheng Sun, Qihua Huang, Guangchao Gai, Zicheng Wu, Wei Meng, Xincheng Chen

**Affiliations:** ^1^Department of Neurosurgery, Xinyi People's Hospital, Xinyi, Jiangsu, China; ^2^Department of Neurosurgery, Xuzhou Central Hospital, 199 Jie Fang Nan Road, Xuzhou 221009, China; ^3^Department of Neurosurgery, The First Affiliated Hospital of Nanjing Medical University, Nanjing, Jiangsu, China

## Abstract

Peroxisome proliferator-activated receptor *α* (PPAR*α*) is a member of the nuclear hormone receptor superfamily and functions as a transcription factor. Previous work showed that PPAR*α* plays multiple roles in lipid metabolism in tissues such as cardiac and skeletal muscle, liver, and adipose tissue. Recent studies have discovered additional roles for PPAR*α* in cell proliferation and metabolism, as well as tumor progression. PPAR*α* is aberrantly expressed in various cancers, and activated PPAR*α* inhibits the proliferation of some tumor cells. However, there have been no studies of PPAR*α* in human gliomas. Here, we show that PPAR*α* is expressed at lower levels in anaplastic gliomas and glioblastoma multiforme (GBM) tissue compared with low-grade gliomas tissue, and low expression is associated with poor patient prognosis. PPAR*α* activates transcription of dynamin-3 opposite strand (DNMO3os), which encodes a cluster of miR-214, miR-199a-3p, and miR-199a-5p microRNAs. Of these, miR-214 is transcribed at particularly high levels. PPAR*α*-induced miR-214 expression causes downregulation of its target E2F2. Finally, miR-214 overexpression inhibits glioma cell growth in vitro and in vivo by inducing cell cycle arrest in G0/G1. Collectively, these data uncover a novel role for a PPAR*α*-miR-214-E2F2 pathway in controlling glioma cell proliferation.

## 1. Introduction

Glioma is the most devastating form of brain cancer and has a 5-year survival rate of <10% and a median survival time of 12–18 months after diagnosis [1–4]. Although modern treatments, including surgery, chemotherapy, and radiotherapy, can improve the prognosis of many cancers, this has not proven to be the case for glioma [5, 6]. Gene therapy is gaining interest as a potential treatment for glioma, but there is a need to identify novel molecular targets.

Peroxisome proliferator-activated receptor *α* (PPAR*α*) was first discovered in* Xenopus* and is a member of the nuclear hormone receptor superfamily [7]. PPAR*α* promotes transcription of the dynamin-3 gene opposite strand (DNMO3os), which encodes a miR-214 and miR-199 cluster [8–10]. MicroRNAs (miRNAs) are endogenous noncoding RNAs with regulatory function in eukaryotes [11]. The mature miRNA recognizes its target mRNAs by complementary base-pairing with the 3′-untranslated region (UTR) of the mRNA and directs it to a multiprotein silencing complex that degrades the mRNA or suppresses its translation, depending on the degree of complementarity [11]. MiR-214 regulates tumor progression by targeting mRNAs encoding proteins such as poly(rC) binding protein-2 (PCBP2), E2F transcription factor 2 (E2F2), and the SUMO-conjugating enzyme UBC9 [12–14]. The finding that E2F2 plays an important role in regulation of the cell cycle is of particular interest for our investigation.

Here, we investigated the function of PPAR*α* in human glioma cells. PPAR*α* inhibited cell proliferation by arresting the cells in the G0/G1 phase of the cell cycle. Overexpression of PPAR*α* in glioma cells promoted transcription of DNMO3os, leading to increased expression of miR-214. Overexpression of miR-214 reduced E2F2 protein expression and inhibited cell proliferation. Our results thus suggest that PPAR*α* inhibits human glioma cell proliferation through a miR-214- and E2F2-dependent pathway and identify novel potential molecular targets for the treatment of human gliomas.

## 2. Materials and Methods

### 2.1. Antibodies

Antibodies were purchased as follows: anti-PPAR*α* was from Abcam (ab215270, Cambridge, UK), anti-E2F2 was from Santa Cruz Biotechnology (sc-633, Dallas, TX, USA), and antiglyceraldehyde 3-phosphate dehydrogenase (GAPDH) was from Cell Signaling Technology (#5174, Boston, MA, USA). Secondary goat anti-mouse IgG and goat anti-rabbit IgG antibodies were from Millipore (401211, 401353, Billerica, MA, USA).

### 2.2. Patient Samples

We obtained clinicopathological data from 158 glioma patients from the Chinese Glioma Genome Atlas (CGGA) data portal (http://cgga.org.cn/). Data were obtained from 48 patients with astrocytoma (A), 13 with oligodendroglioma (O), 8 with anaplastic astrocytoma (AA), 10 with anaplastic oligodendroglioma (AO), 15 with anaplastic oligoastrocytoma (AOA), and 64 with glioblastoma multiforme (GBM). A and O are classified as low-grade gliomas (LGG, WHO Grade II); AA, AO, and AOA are anaplastic gliomas (AG, WHO Grade III), and GBM is WHO Grade IV.

### 2.3. Cell Culture

The human brain glioma cell lines U251 and U87 and the HEK293T cell line were purchased from the cell bank of the Chinese Academy of Sciences (Shanghai, China). U251 and U87 were cultured in DMEM medium (Invitrogen, Carlsbad, CA, USA), and HEK293T was cultured in MEM medium (Invitrogen) supplemented with 10% fetal bovine serum (Gibco, USA). All cells were maintained at 37°C in a 5% CO_2_ atmosphere.

### 2.4. Lentiviral Constructs

PPAR*α* cDNA was cloned into the GFP-expressing pWPXLd plasmid using* Bam*HI and* Pac*I restriction sites, and miR-214 cDNA was cloned into the pGLV3/H1 plasmid using* Bam*HI and* Mlu*I sites. The plasmids and core packaging plasmids were transfected into HEK293T cells using PolyJet (SignaGen, Gaithersburg, MD) for lentivirus production.

### 2.5. Establishment of Stable Glioma Cell Lines

Lentiviruses expressing control protein (GFP), GFP-PPAR*α*, control miRNA, or miR-214 were added to U87 and U251 cells. After 12 hours, the medium was replaced and the cells were cultured for an additional 72 h. The cells were then placed in medium containing 2.5 *μ*g/mL puromycin to select for stable cell lines.

### 2.6. Quantitative Real-Time PCR (RT-PCR)

MiRNA expression was assessed by qRT-PCR. Total RNA was extracted using TRIzol reagent (Invitrogen) and reverse-transcribed using the Quant One-Step RT-PCR Kit (Tiangen, Beijing, China). PCR was performed using an ABI 7300 real-time PCR instrument (Applied Biosystems, Carlsbad, CA, USA) and FastStart Universal SYBR Green Mix (Roche, Basel, Switzerland). Expression levels were calculated using the 2^−ΔΔCt^ method.

### 2.7. Cell Cycle Analysis by Flow Cytometry

Cells were collected, resuspended in 300 *μ*L of phosphate-buffered saline (PBS) plus 700 *μ*l of anhydrous ethanol, and incubated on ice for 15 min. Cells were washed once in PBS, resuspended in 400 *μ*L of PBS, and mixed with 20 *μ*L of 1 mg/mL RNase at 37°C for 30 min. Cells were washed again, resuspended in 500 *μ*L PBS, and mixed with 300 *μ*L of 500 *μ*g/mL propidium iodide for 30 min in the dark. Finally, cell cycle analysis was performed by gating on GFP-positive cells using flow cytometer (BD Biosciences, Franklin Lakes, NJ, USA).

### 2.8. Cell Counting Kit-8 Proliferation Assay

Cells were seeded at 5 × 10^3^ cells/well in 96-well plates and incubated overnight. The following day, 100 *μ*L of medium plus 10 *μ*L CCK-8 kit reagent [2-(2-methoxy-4-nitrophenyl)-3-(4-nitrophenyl)-5-(2,4-disulfophenyl)-2*H*-tetrazolium] (Dojindo Laboratories, Japan) was added to each well and the cells were cultured for 4 h. The optical density at 450 nm was then measured using a SynergyMx MultiMode Microplate Reader (Biotek, Winooski, VT, USA).

### 2.9. Western Blot Analysis

Cells were lysed and equal amounts of protein per sample were resolved by 10% SDS-PAGE. Proteins were transferred to PVDF membranes (Millipore), and the membranes were blocked by incubation with 5% nonfat milk powder for 2 h. Primary antibodies against PPAR*α*, E2F2, or GAPDH were added and the membranes were incubated at 4°C overnight. After washing, the membranes were incubated with the appropriate secondary antibody at room temperature for 2 h. Bound antibodies were revealed using a Pierce ECL Plus Western Blotting Substrate (ThermoFisher Scientific) and imaged using a Bio-Rad ChemiDoc Touch imaging system (Bio-Rad, Hercules, CA, USA). Densitometry analysis of images was compiled with ImageJ software (National Institutes of Health, Bethesda, MD, USA). Protein levels were quantified by densitometry and normalized against GAPDH levels in the same sample.

### 2.10. Luciferase Reporter Assay

The wild-type (WT) 3′-UTR sequence of E2F2 was cloned into pmirGLO vector (GenePharma, Shanghai, China) using* Sac*I and* Xbo*I restriction sites to produce pmirGLO-WT-E2F2-Luc. A mutant vector (pmirGLO-MT-E2F2-Luc) was constructed by deleting the miR-214 binding site (CCUGCUG) in the WT E2F2 3′-UTR. Glioma cells were cotransfected with the WT or MT plasmids and a* Renilla* luciferase plasmid phRL-TK for normalization. Fluorescence levels were detected with the Dual-Luciferase Reporter Assay System (Promega, Madison, WI, USA).

### 2.11. Nude Mouse Tumor Xenografts

Six-week-old female BALB/c nude mice (*n* = 6/group) were obtained from Charles River Company (Beijing, China). Experiments were performed as previously described [15]. In brief, stably infected U87/Control or U87/miR-214 cell lines (2 × 10^6^ cells/0.1 mL) were injected subcutaneously into the upper-left quadrant of the dorsal skin of nude mice. After 8 weeks, the mice were sacrificed and the tumor size was measured. Tumor samples were also homogenized for western blot analysis of E2F2 protein levels.

### 2.12. Statistical Analysis

Patient survival was analyzed using the Kaplan–Meier method. Statistical significance was assessed by Student's *t*-test and one-way ANOVA using SPSS v13.0 software (SPSS Inc., Chicago, IL, USA). Data are presented as the mean ± standard error (SE), and *P* < 0.05 was considered statistically significant.

## 3. Results

### 3.1. PPAR*α* Expression Is Reduced in Human Glioma Tissues

**Figure 1:**
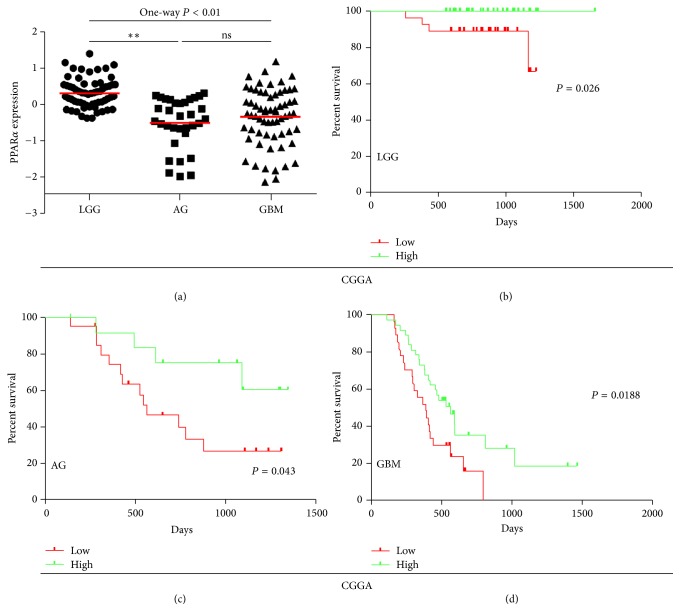
Expression and prognostic significance of PPAR*α* in human glioma tissues. (a) Expression of PPAR*α* in brain tissues from 158 patients with glioma. (b–d) Kaplan–Meier survival curve analysis of the prognostic significance of PPAR*α* expression in patients with (b) low-grade glioma (LGG), (c) anaplastic glioma (AG), and (d) glioblastoma (GBM). According to the expression level of PPAR*α*, it was divided into two groups in LGG, AG, and GBM, respectively, that is, low and high. ^*∗∗*^*P* < 0.01.

To examine the relationship between expression of PPAR*α* in human glioma tissues and patient prognosis, we obtained data on 158 patients from the CGGA portal (http://cgga.org.cn/). Anaplastic gliomas and glioblastoma multiforme (GBM) tissue expressed lower levels of PPAR*α* compared with low-grade gliomas tissue, and expression in gliomas decreased with increasing tumor grade. Thus, PPAR*α* expression decreased in the order LGG (*n* = 61), AG (*n* = 33), and GBM (*n* = 64) (Figure 1(a)). We assessed the prognostic value of PPAR*α* expression levels using Kaplan–Meier survival analysis and discovered a positive relationship between expression levels and the prognosis of LGG, AG, and GBM patients (Figures 1(b)–1(d)). Thus, PPAR*α* is a potential prognostic biomarker in human glioma.

### 3.2. PPAR*α* Inhibits the Proliferation of Glioma Cells

**Figure 2:**
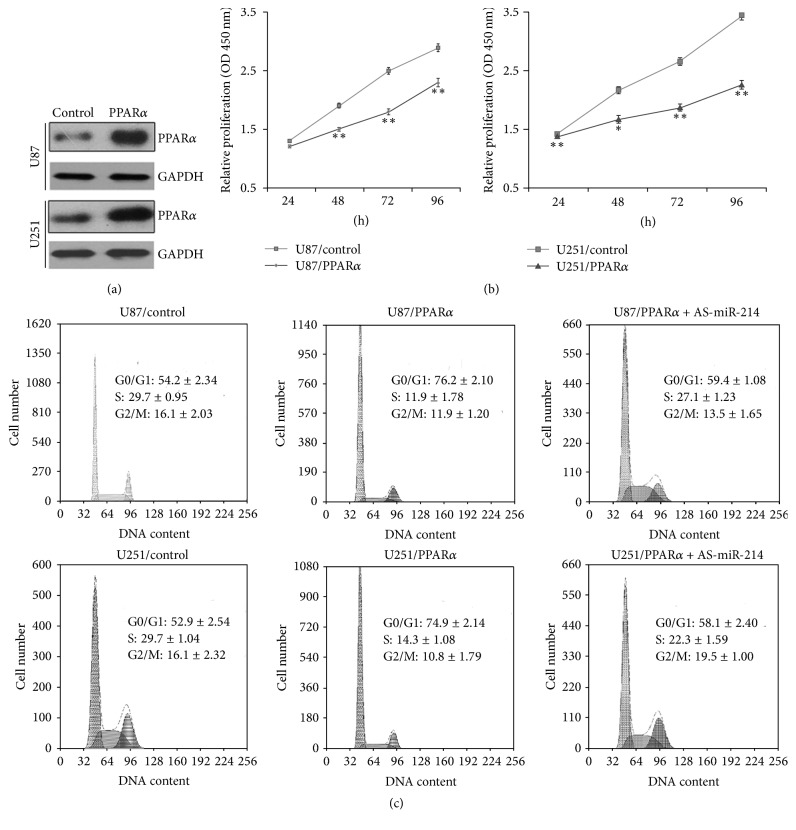
PPAR*α* inhibits proliferation of human brain glioma cells. (a) Western blot analysis of PPAR*α* protein levels in stably infected U87 and U251 cell lines. (b) CCK-8 proliferation experiments. (c) Flow cytometry of distribution of cells in the cell cycle. Experiments were repeated in triplicate; ^*∗*^*P* < 0.05; ^*∗∗*^*P* < 0.01.

To analyze the functional role of PPAR*α* in human glioma, we overexpressed PPAR*α* in two human glioma cell lines, U87 and U251, using lentiviral vectors. Expression levels were verified by western blotting of cell lysates (Figure 2(a)). Cell proliferation was assessed using CCK-8 assays. We found that proliferation was inhibited in cells overexpressing PPAR*α* compared with control cells (Figure 2(b)). To determine how proliferation was blocked, we performed cell cycle analysis by flow cytometry. The results showed that PPAR*α* overexpression caused a block in G1/G0, consistent with the results of the proliferation experiments. Thus, the cell cycle distribution for U87/control cells was 54.2 ± 2.34% (mean ± SE) in G0/G1 phase, 29.7 ± 0.95% in S phase, and 16.1 ± 2.03% in G2/M phase. In U87 cells overexpressing PPAR*α*, the distribution was 76.2 ± 2.10% in G0/G1 phase, 11.9 ± 1.78% in S phase, and 11.9 ± 1.20% in G2/M phase. For U251/control cells, the corresponding distributions were 52.9 ± 2.54% in G0/G1 phase, 29.7 ± 1.04% in S phase, and 16.1 ± 2.32% in G2/M phase. Finally, for PPAR*α*-overexpressing U251 cells, 74.9 ± 2.14% of cells were in G0/G1 phase, 14.3 ± 1.08% were in S phase, and 10.8 ± 1.79% in G2/M phase (Figure 2(c)). Thus, PPAR*α* overexpression appears to inhibit glioma cell proliferation by inducing G0/G1 phase arrest.

### 3.3. PPAR*α* Promotes Transcription of DNMO3os

**Figure 3:**
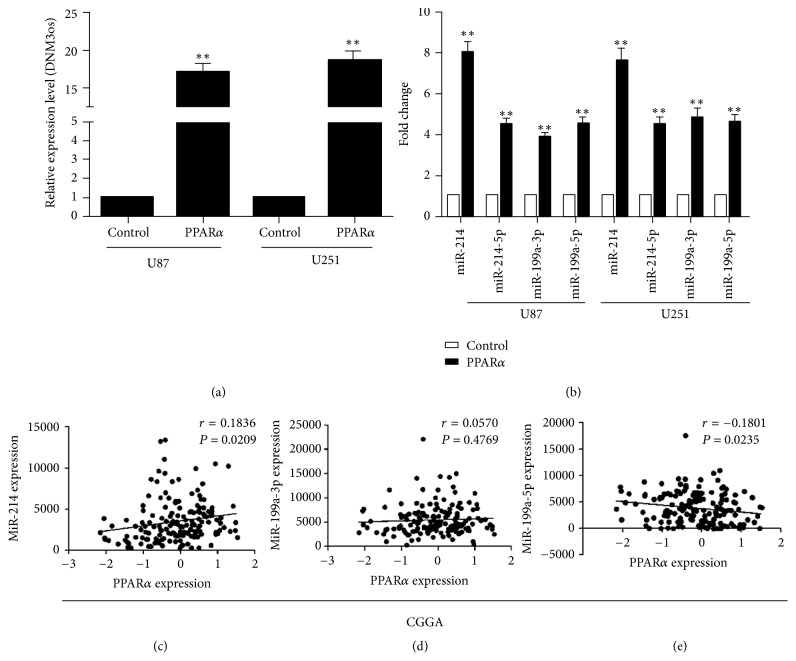
PPAR*α* regulates DNMO3os transcription. (a) PPAR*α* overexpression increases DNMO3os transcription (versus control). (b) Real-time PCR analysis of the expression levels of miR-214, miR-199a-3p, and miR-199a-5p (versus control). (c–e) Correlation analysis between the expression of PPAR*α* and that of miR-214 (c), miR-199a-3p (d), and miR-199a-5p (e). Experiments were repeated in triplicate; ^*∗∗*^*P* < 0.01.

PPAR*α* is known to regulate transcription of the DNMO3os, which encodes miR-199a and miR-214 [8, 16]. To determine whether the inhibition of proliferation induced by PPAR*α* overexpression was related to miR-199a and miR-214 transcription, we examined the activity of DNMO3os in control and PPAR*α*-overexpressing cells. Indeed, basal transcription level was induced in U87 and U251 cells after overexpression of PPAR*α* (Figure 3(a)), and consistent with this, miR-214, miR-199a-3p, and miR-199a-5p levels were higher in PPAR*α*-expressing cells than in the control cells (Figure 3(b)). MiR-214 was the most significantly upregulated miRNA in PPAR*α*-overexpressing cells, and correlation analysis showed a positive association between miR-214 and PPAR*α* levels (Figures 3(c)–3(e)). Collectively, these data suggest that PPAR*α* is involved in the regulation of miR-214 transcription.

### 3.4. MiR-214 Inhibits the Proliferation of Glioma Cells by Targeting E2F2

**Figure 4:**
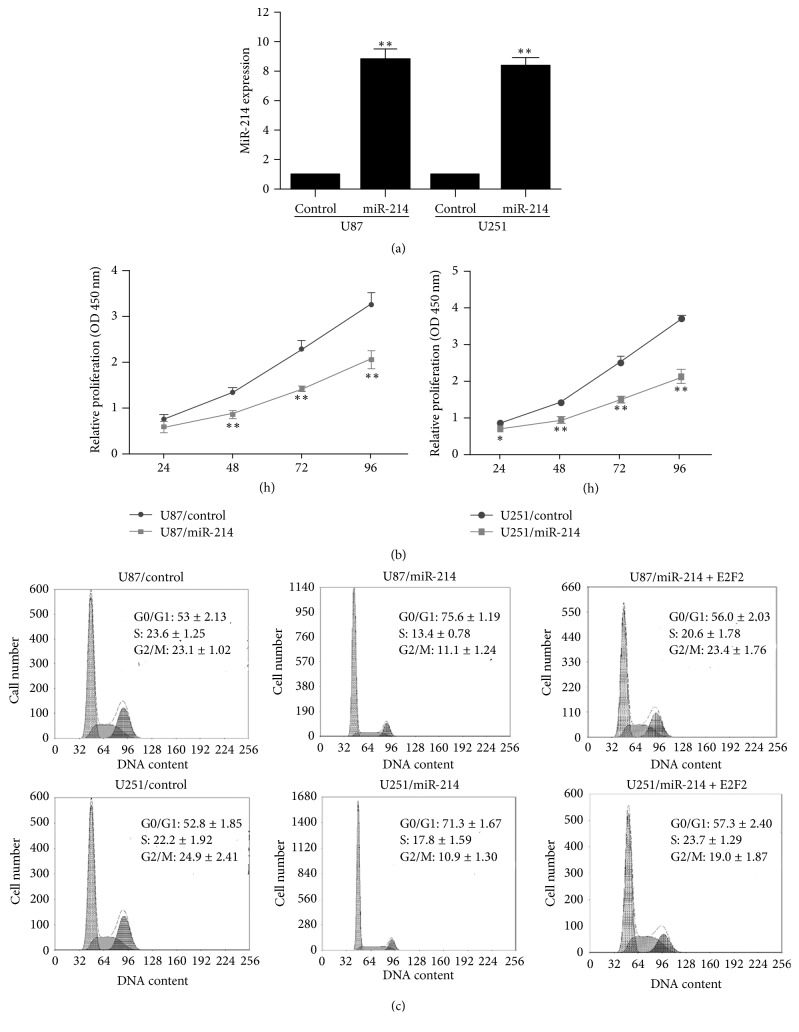
MiR-214 inhibits the proliferation of human glioma cells. (a) Real-time PCR analysis of miR-214 expression in stably infected cell lines (versus control). (b) CCK-8 assay of proliferation of stable cell lines (versus control). (c) Flow cytometric analysis of the cell cycle in stable cell lines. Experiments were repeated in triplicate; ^*∗*^*P* < 0.05; ^*∗∗*^*P* < 0.01.

To determine whether PPAR*α*-mediated transcription of miR-214 is involved in the reduced proliferation of PPAR*α*-overexpressing human glioma cells, we first investigated the effect of lentiviral-mediated overexpression of miR-214. We selected stably infected U87 and U251 cell lines and verified the expression of miR-214 by quantitative real-time PCR (Figure 4(a)). CCK-8 assays demonstrated that miR-214 overexpression not only inhibited the proliferation of U87 and U251 cells (Figure 4(b)) but also increased the proportion of cells in G0/G1, as was observed in PPAR*α*-overexpressing cells. Thus, the proportion of cells in various phases of the cell cycle was as follows: U87/control cells: 53.3 ± 2.13% in G0/G1, 23.6 ± 1.25% in S, and 23.1 ± 1.02% in G2/M; U87/miR-214 cells: 75.6 ± 1.19% in G0/G1, 13.4 ± 0.78% in S, and 11.1 ± 1.24% in G2/M; U251/control cells: 52.8 ± 1.85% in G0/G1, 22.2 ± 1.92% in S, and 24.9 ± 2.41% in G2/M; and U251/miR-214 cells: 71.3 ± 1.67% in G0/G1, 17.8 ± 1.59% in S, and 10.9 ± 1.30% in G2/M (Figure 4(c)).

**Figure 5:**
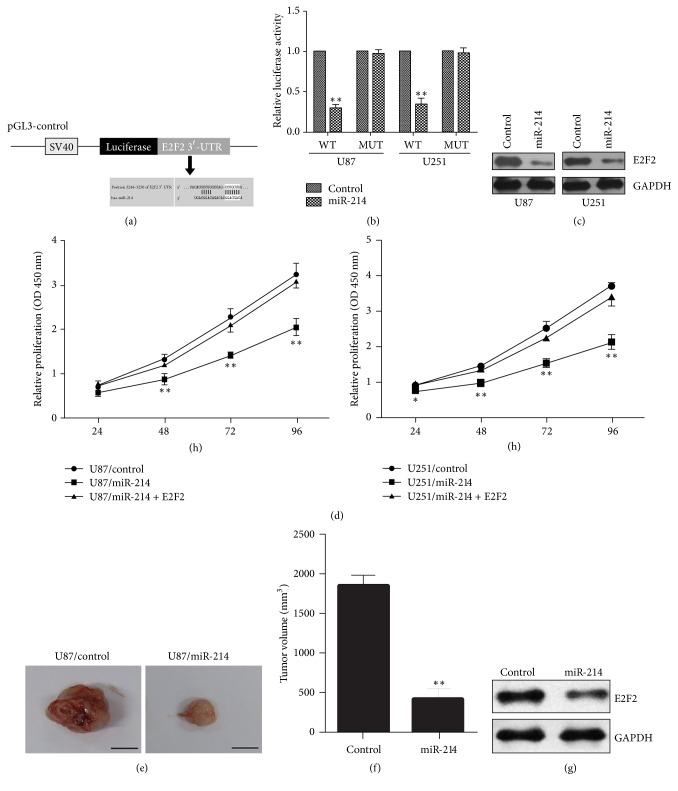
MiR-214 inhibits their tumorigenesis in nude mice via suppression of E2F2. (a) Bioinformatics prediction of miR-214 binding to the 3′-UTR of E2F2. (b) Luciferase reporter assay showed that miR-214 targets the 3′-UTR of E2F2 (versus control). (c) Western blot analysis of E2F2 protein levels. (d) CCK-8 assay of proliferation of U87/miR-214 cells after transfection with an E2F2 expression plasmid. (e) Tumorigenic capacity of stable cell lines in nude mice. (f) Statistical analysis of tumor volumes. (g) Western blot analysis of E2F2 protein expression in tumors excised from nude mice. Experiments were repeated in triplicate; ^*∗*^*P* < 0.05; ^*∗∗*^*P* < 0.01.

To identify potential mRNA targets of miR-214, we performed bioinformatic analysis using TargetScan and PicTar algorithms, which predict target mRNA based on complementarity between miRNA seed sequences and the mRNA 3′-UTR sequences. This analysis identified E2F2 mRNA as a putative target of miR-214 (Figure 5(a)). To confirm this, we performed luciferase reporter assays in human glioma cells expressing luciferase driven by WT human E2F2 3′-UTR or a mutant construct in which the miR-214 binding site is deleted. The cells were cotransfected with control or miR-214 overexpression plasmids. These experiments showed that miR-214 expression could suppress luciferase activity driven by the WT E2F2 3′-UTR but not by the mutant 3′-UTR, confirming that E2F2 is a direct target of miR-214 (Figure 5(b)). Consistent with this, cells overexpressing miR-214 showed reduced expression of E2F2 at the protein level compared with cells expressing a control miRNA (Figure 5(c)). Notably, overexpression of E2F2 partially restored the proliferation of miR-214-overexpressing cells, suggesting that the miR-214 effect was mediated by E2F2 (Figure 5(d)). To determine whether miR-214 overexpression inhibited the growth of glioma cells in vivo as well as in vitro, we injected nude mice subcutaneously with U87 cells expressing either miR-214 or a control miRNA and analyzed tumor growth after 8 weeks. As shown in Figures 5(e)-5(f), tumor growth was reduced by miR-214 overexpression. Western blot analysis of the excised tumors showed that E2F2 expression was lower in tumors derived from miR-214-expressing cells compared with control cells, as expected (Figure 5(g)). These results confirmed that E2F2 is a target of miR-214 in the human glioma cells U87 and U251 and that miR-214 overexpression inhibits tumor growth in vivo.

### 3.5. PPAR*α* Inhibits the Proliferation of Human Glioma Cells via MiR-214-Mediated Regulation of E2F2

**Figure 6:**
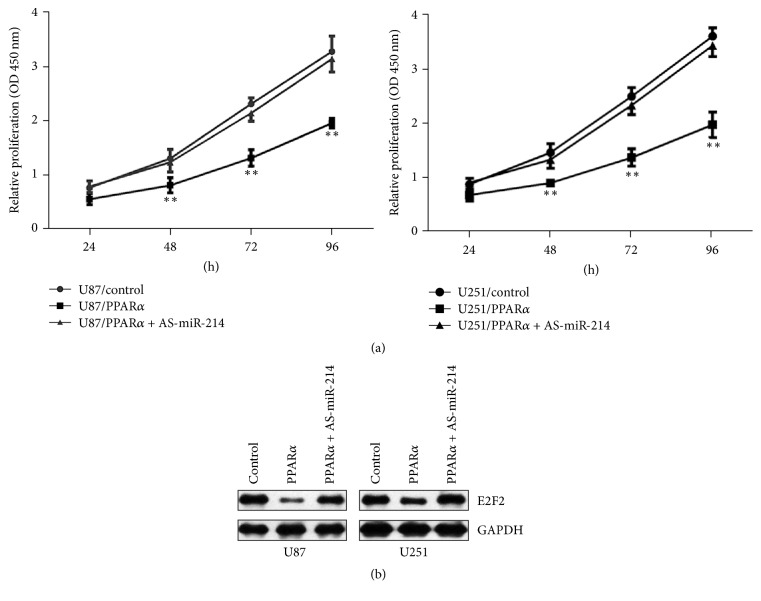
AS-miR-214 restores proliferation of glioma cells overexpressing PPAR*α*. (a) CCK-8 assay of the proliferation of PPAR*α*-overexpressing glioma cells cotransfected with AS-miR-214. (b) Western blot analysis of E2F2 protein expression in AS-miR-214-transfected PPAR*α*-overexpressing glioma cells. Experiments were repeated in triplicate; ^*∗∗*^*P* < 0.01.

Having shown that PPAR*α* promotes miR-214 transcription and that miR-214 overexpression reduces E2F2 protein levels in glioma cells, we next asked whether the inhibitory effects of PPAR*α* on proliferation were mediated through miR-214 and E2F2. To test this, we examined cells transfected with miR-214 antisense (AS-miR-214). Importantly, glioma cell proliferation (Figure 6(a)) and E2F2 protein expression (Figure 6(b)) were both increased in PPAR*α*-overexpressing cells infected with AS-miR-214 compared with PPAR*α* sequence. AS-miR-214 also decreased the proportion of cells in G0/G1 phase of the cell cycle (Figure 2(c)), consistent with the improved cell proliferation. In U87/control cells, 59.4 ± 1.08% of cells were in G0/G1, 27.1 ± 1.23% were in S, and 13.5 ± 1.65% were in G2/M; for U87/AS-miR-214 cells, the corresponding numbers were 58.1 ± 2.40% in G0/G1, 22.3 ± 1.59% in S, and 19.5 ± 1.00% in G2/M. Taken S that PPAR*α* inhibition of glioma cell growth is mediated by the sequential effects on miR-214 transcription and E2F2 expression.

## 4. Discussion

Here, we found that the proliferation of human glioma cells is inhibited by PPAR*α* overexpression, which promotes transcription of DNMO3os, thereby increasing miR-214 levels and consequently decreasing translation of its target E2F2 mRNA. Consistent with this, miR-214 overexpression inhibited cell proliferation by targeting E2F2. Analysis of patient samples from the CGGA database showed that expression of PPAR*α* is low in glioma tissues and that the expression correlated with patient prognosis. Collectively, our findings suggest that PPAR*α* plays an important role in the proliferation of human glioma cells.

Previous studies found that PPAR*α* plays an important role in lipid metabolism, including lipogenesis and lipid catabolism, as well as in insulin resistance, glucose homeostasis, inflammatory responses, and blood pressure regulation [17–19]. Further studies indicated that lipid signaling is involved in cell proliferation, apoptosis, survival, and migration [20]. We previously showed that PPAR*γ*, another member of the PPAR superfamily, is involved in tumor progression [21, 22]. Here, we asked whether and how PPAR*α* might participate in the development of glioma. We found that PPAR*α* expression is low in glioma tissue compared with normal brain tissue and that overexpression of PPAR*α* in glioma cell lines inhibits their proliferation. Related studies have found that PPAR*α* promoted the transcription of the DNMO3 complementary strand [8, 16], which we also observed here in human glioma cell lines.

The DNMO3os transcription unit includes miR-199a and miR-214 [23]. Correlation analysis showed that PPAR*α* have a positive correlation with miR-214. However PPAR*α* do not have correlation with miR-199a-3p and negative correlation with miR-199a-5p. The mechanism that the correlation between PPAR*α* and miR-199a was not clear, which need the future study. Wang et al. found that miR-214 inhibits the proliferation of hepatocellular carcinoma cells by reducing E2F2 mRNA levels [10]. E2F is a group of genes that codifies a family of transcription factors (TF) in higher eukaryotes. The human family of E2F transcription factors consists of eight members, namely, E2F1–E2F8. Suzuki found that knockout of E2F2 could inhibit the proliferation and tumorigenicity of human embryonic stem cells [24]. Our study extends these findings by showing that miR-214 targeting of E2F2 inhibits the proliferation of human brain glioma cells through a PPAR*α*-regulated pathway.

Our data suggest that PPAR*α* expression correlates with the prognosis of glioma patients and inhibits the proliferation of human glioma cells by regulating DNMO3os transcription. We also demonstrated that expression of miR-214, which is encoded by the DNMO3os transcription unit, correlated positively with PPAR*α* levels and that miR-214 inhibits E2F2 expression. Thus, we have elucidated a PPAR*α*-miR-214-E2F2 signaling pathway in human glioma cells. These findings may prove to be helpful for the discovery of new treatments for glioma patients.
